# Different Composite Veneer Surface Preparations for Metal Orthodontic Bracket Bonding: An In Vitro Study

**DOI:** 10.1155/ijod/2175748

**Published:** 2025-01-17

**Authors:** Maryam Omidkhoda, Maryam Hosseini Rivandi, Hamideh Sadat Mohammadipour, Mahboobe Dehghani

**Affiliations:** ^1^Dental Materials Research Center, Mashhad University of Medical Sciences, Mashhad, Iran; ^2^Department of Orthodontics, School of Dentistry, Mashhad University of Medical Sciences, Mashhad, Iran; ^3^School of Dentistry, Mashhad University of Medical Sciences, Mashhad, Iran; ^4^Department of Cosmetic and Restorative Dentistry, School of Dentistry, Mashhad University of Medical Sciences, Mashhad, Iran; ^5^Dental Research Center, Mashhad University of Medical Sciences, Mashhad, Iran

**Keywords:** G-Premio Bond, shear bond strength, surface preparations, veneer composites

## Abstract

**Objective:** This research aimed to assess the shear bond strength (SBS) of metal brackets bonded to composite veneers using different surface preparations.

**Methods:** One-hundred composite disks were divided into 10 different groups whereby each group combines a surface preparation (roughening or no roughening), etching agent (37% phosphoric or 9.5% hydrofluoric acid), adhesive protocol (self-etch or total-etch), and bonding agent (with or without G-Premio Bond). Orthodontic metal brackets were bonded to prepared composite surfaces by Transbond XT resin and light-cured. The prepared samples were incubated at 37˚C for 24 h and underwent thermocycling. A universal testing machine was loaded until the failure moment to assess SBS. Adhesive remnant index (ARI) was used to classify the failure sites between the composite surface and bracket base. Tukey, Fisher's exact, and chi-square tests were used for statistical analyses. Statistical significance level was 0.05.

**Results:** Surface roughening and the type of material used for surface preparation significantly affected bond strength (*p*  < 0.0001). There was no significant difference in bond strength between phosphoric acid and hydrofluoric acid (*p*  > 0.05). The highest bond strength was observed in the group with roughening + total-etch with phosphoric acid + G-Premio Bond (10.24 ± 2.99 MPa). The lowest bond strength was found in the group without roughening, etched with phosphoric acid (1.47 ± 1.06 MPa). In the groups without roughening, ARI scores of 0 and 1 were found, while in the groups with roughening, ARI scores of 0, 1, and 4 were observed.

**Conclusions:** The SBS with G-Premio Bond is high with ARI score 4 which may pose a risk of damage to the veneers during debonding. Therefore, surface preparation of the veneers that result in lesser bond strength should be used unless in patients with frequent bracket breakages.

## 1. Introduction

Presently, we are witnessing an increasing trend toward seeking out orthodontic treatments, especially in the adult population [1, 2]. A vast number of clients have teeth restored with different restorative materials such as amalgam, porcelain, and composite resin [3]. The prevalent use of these restorative materials has created several problems for orthodontists and in bond failure cases, loose bracket replacement is time and money consuming for both the patient and orthodontist [4]. However, in recent years, because of the emergence of new techniques and materials, an acceptable bond strength has been introduced [5].

Although ceramic veneers have superior longevity compared to direct composite veneers, direct resin composite veneers are lower in cost and require less time, which appeals to both patients and clinicians [6]. In addition, they require minimal preparation and can be completed in one session which means reduce the chair time and cost of treatment. So, the resin composite veneers are more selected by the patients, especially in our region. On the other hand, the ceramic veneers are prepared easily by the HF and silane primers and there is no challenge for bonding to ceramic surface, whereas the bonding to aged resin composites is challenging. Many of the orthodontists faced with the debonding of the metal brackets from the previous resin composite veneers. Since the number of patients with previous composite veneers who need the orthodontic treatment is increasing, it may be a big challenge how to increase the bonding to old resin composite.

Studies have found that as composite restorations age, the available vinyl groups for cross-polymerization are reduced; thus, the bonding between the aged and fresh resin composite is decreased [6]. For this reason, different surface treatments have been developed to strengthen this bonding [7]. It has been shown that acid etching has only a cleansing effect and does not affect surface topography [8]. Thus, the increased surface roughness of the old composite helps to produce better interlocking and chemical bonding. In this regard, usually, mechanical and chemical treatments are needed to prepare the surface for bracket attachment [9].

Since the introduction of the acid-etching technique by Buonocore [10] and its usage in bracket bonding by Newman [11], different adhesive methods have been developed. Most of the new methods are faster and easier than the past methods. Self-etching adhesive materials are among these new adhesives that reduce the bonding steps by eliminating priming and etching processes. These materials are a combination of priming and conditioning agents in an acidic solution [12].

The shear bond strength (SBS) of different treatment methods has been investigated on amalgam [13], porcelain [14], and ceramic systems [15]; however, few studies have been conducted on composite resins [2]. G-Premio Bond is a universal, eighth-generation bonding agent that is compatible with total-etch, self-etch, and selective etch techniques, providing excellent versatility. It is perfectly adapted to all direct restorations and can be used to repair indirect restorations without the use of a primer. G-Premio Bond is used with a silane to repair glass or hybrid ceramic and is preferred for hypersensitivity. No studies have been conducted on G-Premio Bond in bonding to veneer composite or porcelain until now.

Our study aimed to assess the SBS of metal brackets attached to composite veneer surfaces using different surface treatments.

## 2. Material and Method

The research protocol of this in vitro study was reviewed and approved by the ethical committee of the Mashhad University of Medical Sciences (ethical code number: IR.mums.sd.REC.1394.319).

### 2.1. Sample Size

The two-way assumption and the averages of 16.2 ± 5.4 and 26.3 ± 4.75 were considered to calculate the sample size [5]. Considering the first and second type error levels of 5%, the sample size for each group was determined as 8. Taking into account, the loss of samples and to raise the confidence level, the sample size of each group was increased to 10.

### 2.2. Specimen Preparation

To prepare the specimens, first, a central tooth in the upper jaw was chosen as a sample to make a mold, using the silicone molding material Speedex Putty (Coltene/Whaledent Inc., Ohio, USA). This was done due to the small curve of the central tooth crown mimicking the oral environment. Next, a 100 composite discs with diameters of 2 mm (equivalent to veneer composite thickness) were created. The prepared samples were horizontally mounted in a self-cure acrylic (Unifast Trad; GC, Japan) and the labial surface was completely horizontal.

### 2.3. Aging Process

Finishing and polishing processes were conducted similarly to the oral environment using soft to rough discs and rubbers from the gingival side to the incisal side for 3 s thrice using each of the rubbers. To produce aged composites, 5000 thermocycling (equivalent to 6 months) was imposed, and composites were soaked in separate baths of cold and warm water for 30 s at a temperature between 5˚ and 55˚.

### 2.4. Grouping

The samples were divided into 10 groups of 10 specimens as follows:

Group 1 (roughening + H3PO4 37%) (control group): A diamond bur with a grit of 125–150 µm was used with the least pressure once from the gingival to the incisal side and once from the mesial to distal side to roughen the samples for 3 s to make a coarse surface. Then, the samples were etched with phosphoric acid 37% (H3PO4 37%) (Morvaetch, Morvabon,…) for 30 s and washed and dried for another 30 s. The Transbond XT primer was applied, followed by bracket bonding with Transbond XT (3 M, Unitek) resin. The bracket (Dentaurum, Germany, Discovery, Roth 22, 3.4 mm width) was mounted on the middle one-third of the disc. The surplus cement was removed using explorer and light-curing on all sides (Blue phase 8; Ivoclar Vivadent, Liechtenstein) using a power of 650 Mw/cm^2^ for 20 s.

Group 2 (without roughening + H3PO4 37%) (control group): This group was completely similar to control 1, omitting the roughening.

Group 3 (roughening + HF 9.5% + silane) (control group): The diamond bur with a grit of 125–150 µm was used to roughen the specimens to create a coarse surface. Hydrochloric acid 9.5% (HF 9.5%) (Porcelain Etchant; Bisco, USA) was used for 90 s and then washed and dried. Then, silane (Bis-Silane; Bisco, USA) was used for 60 s according to the manufacturer's recommendations. After using air power for silane solvent evaporation, Transbond XT resin was applied on the base of the bracket and light-cured on all sides (Blue phase 8; Ivoclar Vivadent, Liechtenstein) using a power of 650 mW/cm^2^ for 20 s.

Group 4 (without roughening + HF 9.5% + silane) (control group): This group was completely similar to control 3, omitting the roughening.

Group 5 (roughening + G-Premio Bond) (test group): After using the diamond bur with a grit of 125–150 µm in the middle third of the disc for roughening, G-Premio Bond was superposed with a microbrush on one-layer as a self-etch method. Then, the composite resin (Transbond XT [3 M, Unitek]) was applied to the base of the bracket and folded on the middle one-third. The surplus cement was removed using explorer and light-curing on all sides (Blue phase 8; Ivoclar Vivadent, Liechtenstein) using a power of 650 mW/cm^2^ for 20 s.

Group 6 (without roughening + G-Premio Bond) (test group): This group was completely similar to the experiment 1 group but without surface roughening.

Group 7 (roughening + H3PO4 37% + G-Premio Bond) (test group): After the diamond bur with a grit of 125–150 µm in the middle third of the disc was used for roughening, G-Premio Bond was used with the total-etch method. After using phosphoric acid 37% in the middle third of the disc for 30 s, G-Premio Bond was used and the composite resin was superposed on the bracket base and attached to the middle one-third of the disc. The surplus cement was removed using explorer and light-curing on all sides.

Group 8 (without roughening + H3PO4 37% + G-Premio Bond) (test group): This group was completely similar to the experiment 3 group but without surface roughening.

Group 9 (roughening+ HF 9.5% + silane + G-Premio Bond) (test group): After the diamond bur with a grit of 125–150 µm in the middle third of the disc for roughening was used, HF 9.5% (Porcelain Etchant; Bisco, USA) was used for 90 s on the specimen surface and then washed and dried. Next, silane (Bis-Silane; Bisco, USA) was used for 30 s according to the manufacturer's recommendations. After using air power for silane solvent evaporation, G-Premio Bond was used in a self-etch manner. Then, the composite resin was applied to the base of the bracket and attached to the middle one-third of the disc. The surplus cement was removed using explorer and light-curing on all sides.

Group 10 (without roughening + HF 9.5% + silane + G-Premio Bond) (test group): This group was completely similar to the experiment 5 group but without surface roughening.

### 2.5. SBS Test

**Figure 1:**
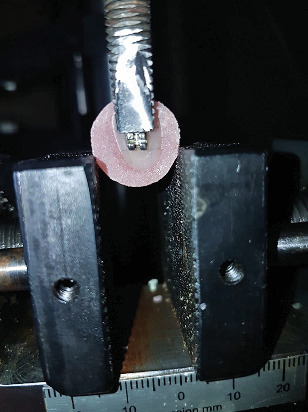
Test of shear bond strength using the universal testing machine.

To assess the SBS, the specimens were loaded using a universal testing machine (Santam, model STM-20, Iran) until failure occurred. The crosshead speed was 0.5 mm/min, according to the previous studies (Figure 1) [16, 17].

### 2.6. Stereomicroscope Assessment

The broken specimens were visualized using a stereomicroscope (Dino lite Pro; Anmo Electronics, Taiwan) with a magnification of 10x. Adhesive remnant index (ARI) was used to classify the failure sites between the composite surface and bracket base. The assessment was according to the Bergland and Artun scoring [18].

Score 0: no adhesive remained on the veneer composite.

Score 1: less than half of the adhesive remained on the veneer composite.

Score 2: more than half of the adhesive remained on the veneer composite.

Score 3: all the adhesive remained on the veneer composite and the bracket mesh was clean.

Score 4: veneer composite was broken.

### 2.7. Statistical Analyses

The two variable variance analysis was used to compare the different materials and roughening, the Tukey test was used to compare the used materials two-by-two, and the Fisher's exact test was used to assess the relation between the type of failure, material, and roughening. Also, the chi-square test was used to assess the amount of remnant adhesive. A *p*-value under 0.05 was considered significant.

## 3. Results

One-hundred veneers were used in the study and equally distributed between 10 groups. The 10 groups were divided into two categories with roughening and without roughening; each containing five subcategories.

### 3.1. Descriptive Data of SBS

The descriptive data of mean SBS are demonstrated in Table 1. Those groups with roughening had a higher SBS (8.90 ± 3.42 vs. 3.14 ± 4.84). Also, the G-Premio Bond total-etch + H3PO4 treatment had the highest SBS among different materials (8.70 ± 2.86). Details of comparison between H3PO4 and HF groups and also between self-etch and total-etch methods are demonstrated in Table 2.

### 3.2. Comparing SBS Regarding the Presence of Roughening or Type of Material

There was a significant difference in the presence or absence of roughening in SBS (*p*  < 0.0001). This difference was also significant for the type of material (*p*  < 0.0001). Furthermore, the interaction between the type of material and roughening was not significant (*p*=0.25).

According to Table 3, regardless of the presence or absence of roughening, there was no significant difference in the bond strength of H3PO4 and HF surface treatment. Also, there was no significant difference in SBS regarding using G-Premio Bond self-etch, G-Premio Bond total-etch with H3PO4, and G-Premio Bond total-etch with HF.

**Figure 2:**
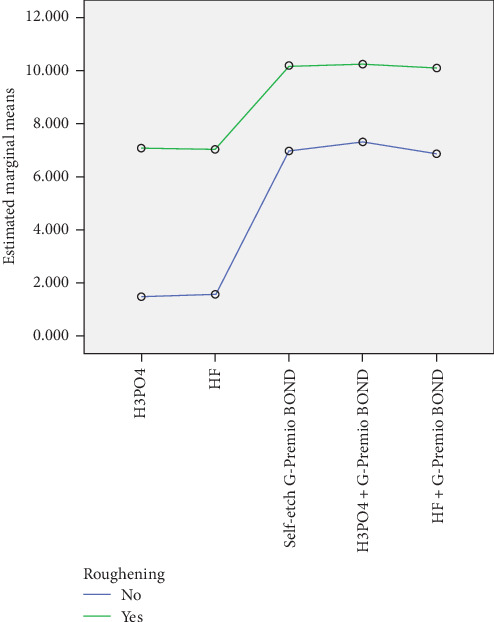
Linear diagram of the shear bond strength of groups with and without surface roughening.

Each of the G-Premio Bonds had higher bond strength compared to the HF or H3PO4 groups (*p*  < 0.05). Table 3 shows the details of these comparisons in a two-by-two manner and the results are shown in Figure 2.

### 3.3. ARI Classification

There was a significant difference in the ARI classification between those with roughening or without roughening (*p*=0.001).

### 3.4. Groups Without Roughening

Only ARI scores of 0 and 1 were found in the groups without roughening. The number of fractures in HF and H3PO4 was significantly lower than in other groups. There was no significant difference between the HF and H3PO4 groups and G-Premio Bond self-etch, G-Premio Bond total-etch with H3PO4, and G-Premio Bond total-etch with HF (*p*  > 0.05). The ARI 0 was significantly lower in the H3PO4 group compared to the G-Premio Bond total-etch group (*p*  < 0.05); however, there was no significant difference between other groups. There was also no notable difference in the number of ARI score of 1 between the groups (*p*  > 0.05).

### 3.5. Groups With Roughening

In this group, only ARI scores of 0, 1, and 4 were found. The ARI score of 0 was significantly higher in the HF group compared to the G-Premio Bond self-etch and G-Premio Bond total-etch with H3PO4 (*p*  < 0.05). Furthermore, numbers of ARI score of 0 were significantly higher in the H3PO4 group compared to the G-Premio Bond total-etch with H3PO4 (*p*  < 0.05). There was no significant difference in the number of ARI score of 1 between the groups (*p*  > 0.05).  Table 4 shows details of the abovementioned results.

## 4. Discussion

Our study aimed to assess the amount of SBS in the surface of the composite veneer surface attached to the metal bracket using different surface treatments. Our results showed that the presence of roughening produced higher bond strength compared to the without roughening group and the type of material independently affected the SBS. This mechanism produces craters and streaks that create better micro- and macro-mechanical attachments. Studies have reported that acid etching with H3PO4 and HF only has a cleansing effect.

Each of the G-Premio Bond groups had higher bond strength compared to the H3PO4 or HF groups. However, there was no significant difference in bond strength between the three G-Premio Bond groups. Also, there was no notable difference in this regard between the HF and H3PO4 groups.

The easiest and fastest method of bond strength assessment used in the studies is the SBS test; thus, we used this test in our experiment [19]. Several factors affect the bonding strength of the bracket to the aged composite resin including composite resin type, adhesive resin thickness, contamination, moisture, bracket base geometry and dimension, aging of the composite, storage conditions, and testing method [17, 20]. Another crucial factor is the kind of surface treatment. The treatment method includes mechanical and chemical conditionings to increase the bonding area and bring better adhesion [21].

The importance of the bond strength of the bracket to the composite veneer is because of forces applied through mastication. Thus, a minimum SBS is needed for an acceptable bracket attachment, which is reported to be in a range of 6–8 MPa [22]. This was only found in the case of surface roughening in our study. Moreover, most of the cases were ARI0 or ARI1, which shows the surface of bond failure is predominantly between the veneer and the adhesive.

Although none of the studies in the literature assessed G-Premio bond brackets, studies on other types of brackets have been conducted. Bayram et al. [2] assessed SBS on the interface of Transbond XT brackets using different surface treatments. They reported that roughening the surface with the bur method can bring the strongest SBS compared to other groups that are significantly higher compared to the absence of surface roughening. They reported that surface treatment with only HF or H3PO4 accompanies insufficient bond strength. Bishara, Ajlouni, and Oonsombat [23] further supported the conclusion that surface roughening brings better SBS, as the bond strength in the groups without roughening was 9.4 MPa, and in groups with roughening, it was 6.1 MPa. Viwattanatipa et al. [24] also reported that surface roughening brings higher bond strength. Our results were in line with all the abovementioned studies in the case of surface roughening.

It is believed that three types of mechanisms are the cause of adhesion of the bracket to the aged composite resin: chemical bonding to the matrix, chemical bonding to the filler particles, and micromechanical retention [25]. Micromechanical retention is achieved by penetration of the monomer components into microcracks in the matrix. Thus, it is believed that surface treatment with roughening brings better bonding by strengthening the micro- and macro-mechanical adhesions by providing deep craters and streaks on the surface of the composite resin [26]. This roughening is produced by different instruments and methods including the diamond bur and sandblasting method. Our study used the diamond bur for roughening. Furthermore, it has been demonstrated that acid etching only has a cleansing effect and does not affect the composite surface [27].

The most important use of self-etch adhesives was the absence of hyperdecalcification and the useless amount of etching that occurs with H3PO4 surface treatment [28]. The self-etch adhesives do not provide usual micro or macrotags; thus, the dental surface has a lower ability to be smeared with the composite compared to the total-etch composites, reducing the risk of damage to the enamel during the debonding process [23]. The SBS of the self-etch group was 6.79 MPa, which was lower than the total-etch group with 7.32 MPa; however, it was in a clinically acceptable range. Regardless of the presence or absence of roughening, the self-etch and total-etch groups had higher SBS compared to the HF and H3PO4 groups, which were in line with the previous studies [28, 29].

Although our study was the first in its field, it faced some limitations. One of these limitations was the rarity of healthy central teeth; thus, we used composite veneers mounted on acryl, and due to the absence of chemical bonding with acryl, the SBS was lower compared to real tooth enamel. Thus, in groups with strong bonding of the bracket to the veneer composite, during the debonding process, the composite veneer was broken and an ARI4 was recorded. Another limitation of this study is regarding the research design, it is an in vitro study whereby findings may not fully translate to a clinical setting where other factors, such as occlusal forces, moisture, and saliva can affect bonding strength.

## 5. Conclusions

This study was conducted on G-Premio Bond for orthodontic bracket bonding to compsite veneer using different surface treatments. The current study showed that surface roughening provided better bracket adhesion and higher SBS. According to current study, the SBS with G-Premio Bond is high with ARI score 4 which may pose a risk of damage to the veneers during debonding. Therefore, surface preparation of the veneers that result in lesser bond strength should be used unless in patients with frequent bracket breakages.

## Figures and Tables

**Table 1:** Mean shear bond strength of different surface treatments with their frequency.

Roughening	Surface treatment	Mean SBS ± SD (MPa)	Frequency
Without roughening	H3PO4	1.47 ± 1.06	10
	HF	1.57 ± 1.11	10
	Self-etch G-Premio Bond	6.97 ± 1.75	10
	Total-etch with H3PO4, G-Premio Bond	7.32 ± 1.98	10
	Total-etch with HF G-Premio Bond	6.86 ± 1.90	10
	Total	3.14 ± 4.84	50

With roughening	H3PO4	7.08 ± 4.15	10
	HF	7.04 ± 2.18	10
	Self-etch G-Premio Bond	10.19 ± 2.08	10
	Total-etch with H3PO4, G-Premio Bond	10.24 ± 2.99	10
	Total-etch with HF G-Premio Bond	10.11 ± 3.92	10
	Total	8.90 ± 3.42	50

Total	H3PO4	4.28 ± 4.12	20
	HF	4.30 ± 3.27	20
	Self-etch G-Premio Bond	8.58 ± 2.49	20
	Total-etch with H3PO4, G-Premio Bond	8.70 ± 2.86	20
	Total-etch with HF G-Premio Bond	8.48 ± 3.43	20
	Total	6.85 ± 3.85	100

**Table 2:** Comparing H3PO4 and HF groups and self-etch and total-etch methods.

Material	Number	SBS (MPa)	*p*-Value
H3PO4	20	4.28	>0.999
HF	20	4.30	

Self-etch G-Premio Bond	20	8.48	0.999
Total-etch with H3PO4, G-Premio Bond	20	8.58	
Total-etch with HF G-Premio Bond	20	8.70	

**Table 3:** Two-by-two analyses of the used material for surface treatment.

Material	Mean difference	Standard error	Minimum	Maximum	*p*-Value
H3PO4					
HF	−0.023	0.79	−2.24	2.19	>0.999
Self-etch G-Premio Bond	−4.29	0.79	−6.51	−2.07	<0.0001
Total-etch with H3PO4, G-Premio Bond	−4.42	0.80	−6.67	−2.17	<0.0001
Total-etch with HF G-Premio Bond	−4.20	0.79	−6.42	−1.98	<0.0001
HF					
H3PO4	0.023	0.79	−2.19	2.24	>0.999
Self-etch G-Premio Bond	−4.27	0.79	−6.49	−2.05	<0.0001
Total-etch with H3PO4, G-Premio Bond	−4.39	0.80	−6.64	−2.14	<0.0001
Total-etch with HF G-Premio Bond	−4.18	0.79	−6.40	−1.96	<0.0001
Self-etch G-Premio Bond					
H3PO4	4.29	0.79	2.07	6.51	<0.0001
HF	4.27	0.79	2.05	6.49	<0.0001
Total-etch with H3PO4, G-Premio Bond	−0.12	0.80	−2.37	2.12	>0.999
Total-etch with HF G-Premio Bond	0.09	0.79	−2.12	2.31	>0.999
Total-etch with H3PO4, G-Premio Bond					
H3PO4	4.42	0.80	2.17	6.67	<0.0001
HF	4.39	0.80	2.14	6.64	<0.0001
Self-etch G-Premio Bond	0.12	0.80	−2.12	—	>0.999
Total-etch with HF G-Premio Bond	0.21	0.80	−2.03	—	0.999
Total-etch with HF G-Premio Bond					
H3PO4	4.2	0.79	1.98	6.42	<0.0001
HF	4.18	0.79	1.96	6.40	<0.0001
Self-etch G-Premio Bond	−0.09	0.79	−2.31	2.12	>0.999
Total-etch with H3PO4, G-Premio Bond	−0.21	0.80	−2.46	2.03	0.999

**Table 4:** Comparing the adhesive remnant index according to the surface treatment with different materials.

Scratch	Material	ARI 0	ARI 1	ARI 4	Total	*p*-Value
Without roughening, *N* (%)	H3PO4	8 (80%)	2(20%)	0 (0%)	10 (100%)	<0.0001
	HF	6 (66.7%)	3 (33.3%)	0 (0%)	10 (100%)	
	Self-etch G-Premio Bond	2 (20%)	0 (0%)	8 (80%)	10 (100%)	
	Total-etch with H3PO4, G-Premio Bond	1 (11.1%)	0 (0%)	8 (88.9%)	10 (100%)	
	Total-etch with HF G-Premio Bond	2 (20%)	1 (10%)	7 (70%)	10 (100%)	

With roughening, *N* (%)	H3PO4	2 (20%)	1 (10%)	7 (70%)	10 (100%)	0.001
	HF	4 (40%)	1 (10%)	5 (50%)	10 (100%)	
	Self-etch G-Premio Bond	0 (0%)	0 (0%)	10 (100%)	10 (100%)	
	Total-etch with H3PO4, G-Premio Bond	0 (0%)	0 (0%)	10 (100%)	10 (100%)	
	Total-etch with HF G-Premio Bond	0 (0%)	0 (0%)	10 (100%)	10 (100%)	

Total, *N* (%)	H3PO4	10 (50%)	3 (15%)	7 (35%)	20 (100%)	<0.0001
	HF	10 (52.6%)	4 (21.1%)	5 (26.3%)	19 (100%)	
	Self-etch G-Premio Bond	2 (10%)	0 (0%)	18 (90%)	20 (100%)	
	Total-etch with H3PO4, G-Premio Bond	1 (5.3%)	0 (0)	18 (94.7%)	20 (100%)	
	Total-etch with HF G-Premio Bond	2 (10%)	1 (5%)	17 (85%)	20 (100%)	

## Data Availability

Data generated or analyzed during this study are available from the corresponding author upon reasonable request.
